# Single-Nucleotide Variations in Cardiac Arrhythmias: Prospects for Genomics and Proteomics Based Biomarker Discovery and Diagnostics

**DOI:** 10.3390/genes5020254

**Published:** 2014-03-27

**Authors:** Ayman Abunimer, Krista Smith, Tsung-Jung Wu, Phuc Lam, Vahan Simonyan, Raja Mazumder

**Affiliations:** 1 Department of Biochemistry and Molecular Medicine, George Washington University, Washington, DC 20037, USA; E-Mails: aabunimer115@gmail.com (A.A.); ksmith7@gwu.edu (K.S.); sunearth@gwu.edu (T.-J.W.); 2 Center for Biologics Evaluation and Research, Food and Drug Administration, Rockville, MD 20852, USA; E-Mails: phuclam87@gmail.com (P.L.); Vahan.Simonyan@fda.hhs.go (V.S.); 3 McCormick Genomic and Proteomic Center, George Washington University, Washington, DC 20037, USA

**Keywords:** cardiac arrhythmia, SNP, genome-wide association, proteomics, next-generation sequencing, S-nitrosylation, cysteine, nsSNV, biocuration

## Abstract

Cardiovascular diseases are a large contributor to causes of early death in developed countries. Some of these conditions, such as sudden cardiac death and atrial fibrillation, stem from arrhythmias—a spectrum of conditions with abnormal electrical activity in the heart. Genome-wide association studies can identify single nucleotide variations (SNVs) that may predispose individuals to developing acquired forms of arrhythmias. Through manual curation of published genome-wide association studies, we have collected a comprehensive list of 75 SNVs associated with cardiac arrhythmias. Ten of the SNVs result in amino acid changes and can be used in proteomic-based detection methods. In an effort to identify additional non-synonymous mutations that affect the proteome, we analyzed the post-translational modification S-nitrosylation, which is known to affect cardiac arrhythmias. We identified loss of seven known S-nitrosylation sites due to non-synonymous single nucleotide variations (nsSNVs). For predicted nitrosylation sites we found 1429 proteins where the sites are modified due to nsSNV. Analysis of the predicted S-nitrosylation dataset for over- or under-representation (compared to the complete human proteome) of pathways and functional elements shows significant statistical over-representation of the blood coagulation pathway. Gene Ontology (GO) analysis displays statistically over-represented terms related to muscle contraction, receptor activity, motor activity, cystoskeleton components, and microtubule activity. Through the genomic and proteomic context of SNVs and S-nitrosylation sites presented in this study, researchers can look for variation that can predispose individuals to cardiac arrhythmias. Such attempts to elucidate mechanisms of arrhythmia thereby add yet another useful parameter in predicting susceptibility for cardiac diseases.

## 1. Introduction

Cardiac arrhythmias encompass a range of conditions in which the normal rhythm of the heart is disrupted. The conditions include sudden cardiac death (SCD) and atrial fibrillation (AF), the most common form of arrhythmia [1,2]. The causes of arrhythmias such as AF are multifaceted and this presents difficulties in their treatment [3]. The number of Americans afflicted with arrhythmia is expected to increase as the population ages. Consequently, understanding the exact mechanisms of the disease and developing treatments is important.

DNA sequencing has evolved from its beginnings in the Sanger method. Now, next-generation sequencing (NGS) technologies allow the sequencing of millions of fragments of DNA in unison from a single sample. This process of parallel sequencing allows the whole genome to be sequenced in less than a day and is expected to be used in clinics in the near future [4]. Advances in DNA sequencing and genome-wide association studies are impacting detection, management, and treatment of diseases [5,6,7,8]. Several studies have investigated mutations and their associations with cardiac diseases. For example, long QT-syndrome (LQTS) is a disease characterized by prolonged repolarization of the heartbeat [9]. Research has shown that LQTS stems from mutations in genes encoding cardiac ion channels [10]. Over 100 mutations have been identified in 5 separate cardiac ion channel genes [9,10]. Other cardiac channelopathies, or disorders of the heart channel, affect normal cardiac rhythm in individuals [11]. Heritable cardiac arrhythmias, acquired from genetic alterations of these ion channels involved in cardiac heart rhythm, predispose affected individuals to sudden death [11]. Research on sudden cardiac death (SCD), which mainly results from severe ventricular arrhythmias, shows that a majority of mutations are found in the coding regions of ion channel units and key regulatory proteins. These mutations often lead to ion channel dysfunctions or affect biophysical properties of ion channels involved in normal heart function [12]. Further, computer modeling and simulations of cardiac myocytes have enabled scientists to represent variations in gene expression and allows them to reconstruct the effects of mutations characterized by functional changes in proteins [13]. Similar to DNA sequencing, proteomics technologies are also rapidly improving [14]. It is now possible to use proteomic technologies in conjunction with genomic technologies to detect and/or validate nsSNVs [15,16]. Such validation is critical to distinguish between inherited variations, somatic DNA and RNA modifications [17]. Therefore, having a list of amino acid changes that are potentially related to a specific disease can be of immense value as priority targets for proteomic validation. 

One method for studying arrhythmias is investigating the genetic roots of the condition. This report presents the SNVs discovered or confirmed to have associations with arrhythmia over the past twenty years through genome-wide association and replication studies. Table 1 shows ten amino acid changing mutations, which can be detected using modern proteomic technologies [18]. While the variations are contained within a range of genes, the report further investigates two genes in particular. The first is NOS1AP, the most represented gene in Table S1, with 12 SNPs. The second gene is KCNN3, which hosts one of the exonic variants.

**Table 1:** Summary of variant information from Table S1.

Gene	Number of Variations	Chromosome Number	Amino Acid Change	Gene Description
SCN10A	2	3	V1073A	Sodium ion channel
HEY2	1	6	0	Cardiovascular helix-loop-helix factor 1
SCN5A	5	3	S1103Y, H558R	Sodium ion channel
TBX5	1	12	0	T-box transcription factor
NOS1AP	12	1	0	Nitric oxide synthase 1 adaptor protein
ATP1B1	2	1	0	Sodium/potassium-transporting ATPase subunit beta-1
ZFHX3	3	16	0	Zinc finger homeobox protein 3
KCNN3	3	1	N44N	Small conductance potassium channel
KCNJ2	2	17	0	Potassium ion channel
XYLB	3	3	0	Energy metabolism
EXOG	3	3	0	Endonuclease
ACVR2B	3	3	0	Activin Receptor
RNF207	1	1	G603A	Ring finger protein
PLN	2	6	0	Cardiac Muscle
KCNH2	5	7	K897T, K557T	Potassium voltage gated channel
KCNQ1	4	11	G643S	Potassium voltage gated channel
LITAF	1	16	0	DNA binding protein
NDRG4	1	16	0	Mitogenic signalling
AGTR1	1	3	0	Angiotensis II receptor
KNG1	1	3	0	Kininogen
KCNE1	3	21	D85N, S38G	Potassium voltage gated channel
KCNE4	1	2	D196E	Potassium voltage-gated channel

In an attempt to identify more amino acid changing variations potentially connected to the disease, we investigated the post-translational modification (PTM) nitrosylation. S-nitrosylation involves covalent attachment of Nitric Oxide (NO) to the thiol side chain of cysteine to form an S-nitrosothiol (SNO) [19]. NO is a well-known signaling molecule in the cardiovascular system and its role in cardiovascular diseases has been established [20]. S-nitrosylation is a reversible and selective PTM which regulates protein activity, cellular signal transduction, localization, and stability [19,21]. Modified levels of SNO proteins in the blood have been associated with high risks in patients with cardiovascular diseases [19]. Several studies have investigated these associations. Massy *et al*. found elevated plasma SNO levels in patients undergoing chronic hemodialysis predict cardiovascular outcomes [22]. Another study investigated NO as an endocrine vasoregulator in red blood cells and the potential impact NO plays in congestive heart failure [23]. In addition to nitrosylation other PTMs, such as glycosylation and phosphorylation, are also known to maintain the cardiac rhythm [12]. Current studies examine the association between acquired arrhythmias and PTMs of the cardiac sodium channel, which is involved in cardiac action potentials [24]. Cutler *et al*. demonstrated that neuronal nitric oxide synthase (NOS1) inhibition in the intact heart, along with the presence of increased myocardial Ca^2+^, increases an individual’s chances of Ca^2+^-mediated triggered arrhythmias [25]. Based on the aforementioned connections of nitrosylation and heart disease, we analyzed experimental and predicted loss of S-nitrosylation sites that are affected by nsSNVs.

## 2. Experimental

Manual curation of scientific articles retrieved using PubMed database [26] produced the 75 SNPs shown in Table S1. The articles selected from the PubMed database were found using search terms that included “Arrhythmia”, “Genome-wide”, “Association”, “Study”, “Cardiac”, and “SNP”. The articles published in the last 20 years were examined for reports of SNVs associated with cardiac arrhythmias. In these articles, SNVs discovered to have statistically significant associations with cardiac arrhythmias were added to the data shown in Table S1. To ensure consistency, articles that reported SNVs as a result of meta-analysis studies were not included in the list. Only SNVs that were discovered or confirmed in studies by the publishing group were eligible for inclusion. Of those SNVs, only those with statistically significant associations with cardiac arrhythmias were included. After extraction, background information on each SNV was collated from the Single Nucleotide Polymorphism Database or dbSNP [26] (Table 1 and Table S1). This public domain archive hosts a broad collection of simple genetic variations.

The seven experimentally verified S-nitrosylated cysteine sites that are affected by nsSNVs (Table 2) were obtained through mapping of S-nitrosylated proteins to the variations. These S-nitrosylated proteins were obtained from a literature review of endogenously S-nitrosylated proteins by Gould *et al*. [27]. Table 2 displays the proteins affected by the nsSNVs. Prediction of S-nitrosylation sites was performed as follows: the complete human proteome was obtained from UniProtKB/Swiss-Prot and GPS-SNO tool was used to predict N-nitrosylation sites [28]; pairwise alignments between *Homo sapiens* proteins and *Mus musculus*, *Drosophila melanogaster*, *Arabidopsis thaliana* and *Saccharomyces cerevisiae* proteins was performed followed by mapping of nsSNVs from SNVDis [29] to create a table that included predicted S-nitrosylation sites, conserved sites among the species and variation. Enrichment analysis was performed to identify over- or under-represented pathways or GO terms in the protein dataset, compared to their occurrence in the complete human proteome (Table 3). The expected occurrence of a pathway or GO term was calculated based on the actual number of times it was present in the human proteome [29,30]. Over- and under-represented pathways and processes were compared through significance in p values calculated based on methods described earlier [31].

**Table 2:** Loss of experimentally confirmed S-nitrosylation sites by nsSNVs.

Protein name	Position	Variation	Subseq	Ortholog	Species
Alpha-enolase	357	c->y	qackl	P21550	Mouse
Cysteine and glycine-rich protein 3	58	c->g	iyckv	P50462	Mouse
Myosin-6	949	c->y	decse	Q02566	Mouse
Catenin beta-1	619	c->y	vlcel	Q02248	Mouse
Elongation factor 1-alpha 1	234	c->w	ldcil	P10126	Mouse
Myelin proteolipid protein	220	c->y	kvcgs	P60202	Mouse
E3 ubiquitin-protein ligase XIAP	90	c->y	pncrf	A2BGY6	Mouse

**Table 3:** Functional analysis of predicted cysteine S-nitrosylation sites with mutations.

Analysis	Functional object	Observed	Expected	+/−	*p* value
PANTHER Pathways	Blood Coagulation	13	3.82	+	3.64E-29
PANTHER Protein Classification	Receptor (includes G-protein coupled receptor)	190	123.24	+	4.13E-07
	Cytoskeletal protein	98	64.03	+	5.46E-03
	Defense/immunity protein	72	46.53	+	4.48E-02
GO Biological Process	Muscle contraction	73	37.48	+	1.92E-05
	Neurological system process	188	128.53	+	2.04E-05
	Cellular component organization	131	84.15	+	9.63E-05
GO Molecular Function	Receptor activity (includes G-protein coupled receptor activity)	190	124.18	+	6.02E-07
	Motor activity	26	9.52	+	1.05E-03
	Structural constituent of cytoskeleton	98	64.03	+	4.52E-03
GO Cellular Component	Cytoskeleton	98	64.03	+	1.09E-03
	Microtubule	35	17.70	+	5.89E-03
	Intracellular	107	76.44	+	1.36E-02

## 3. Results and Discussion

Table S1 is a resource for researchers or clinicians to quickly identify SNVs associated with arrhythmias. The inclusion of information such as flanking base pairs and exact chromosomal position for each of the variants in Table S1 expedites the process of testing patients if whole genome, targeted sequencing data or proteomics data is available. Below are details of some of the genes and variations associated with them that correlate to arrhythmia.

### 3.1. NOS1AP

As shown in Table 1, the gene with the most variations was NOS1AP. The NOS1AP gene is located on chromosome 1 at q23.3. The gene codes for a regulatory protein—carboxyl-terminal PDZ ligand of neuronal nitric oxide synthase [32]. The protein influences the activity of nitric oxide synthase (NOS). When the NOS1AP protein is expressed in the heart, cardiac repolarization is typically accelerated. The NOS1AP enzyme inhibits l-type calcium channels [33]. The closing of l-type calcium channels inhibits the influx of Ca^2+^ current. Consequently, intracellular calcium concentrations do not increase, and the beta-adrenoreceptor stimulation of the heart is suppressed. This system, starting with the expression of the NOS1AP protein and resulting in a change in the electrophysiology of the heart, helps explain the role of NOS1AP variants with QT interval duration. QT interval is a measure of cardiac repolarization and a common biomarker of arrhythmia. The QT interval is estimated to be 30% heritable [34]. Studies have shown that the noncoding variants in NOS1AP are influential [35]. This is consistent with the data in Table 1 where all of the NOS1AP variants are intronic. The genome-wide association study by Arking *et al*. investigated 200 individuals and found associations in these individuals between QT interval length and the common variants in the noncoding regions of NOS1AP. These findings were replicated in a community of Old Order Amish [36].

Due to the complexity of the biological system, it is difficult to predict how these intronic variants specifically influence the malfunction or mis-regulation of the NOS1AP gene. However, the presence of resources such as Table S1 can help organize further inquiries into the role of intronic variants in disease development.

### 3.2. KCNN3

The SNV rs1131820 is a synonymous SNV found in the KCNN3 gene. The KCNN3 gene is located on chromosome 1q21 and is responsible for coding the protein SK3. The protein SK3 belongs to a family of proteins that operate as calcium activated potassium channels. Specifically, SK3 is a small-conductance calcium activated potassium channel [37]. This indicates that SK3 is voltage insensitive and the opening of the channel is instead reliant on the presence of calcium ions. The protein plays a role after hyper-polarization, a calcium dependent process that is executed after the firing of an action potential in a neuron. A variant in KCNN3 may influence the regulation or function of the SK3 protein. Reduced efficacy of the SK3 protein can inhibit influx of potassium ions through the potassium channel across a membrane. This can disrupt firing of the neuron. Consequently, KCNN3 variants expressed in regions such as the brain or the heart can have significant consequences on those delicate biological systems.

In the heart, a KCNN3 variant was shown to have an association with lone atrial fibrillation. This SNP rs1131820, shown in Table 1 is synonymous. This indicates that there is no consequent change in amino acid from the base pair mutation. One would anticipate that without a change in the amino acid, the effects of a variation would not manifest. However, in this circumstance, a carrier of the two major alleles GG at rs1131820 had an odds ratio of 2.85 (95% CI 1.13–7.18, *p* = 0.026) for lone atrial fibrillation when compared to carriers of the minor allele AA [38]. This suggests that carriers of the two major alleles GG are nearly three times more likely to develop lone atrial fibrillation. It is still unclear how a synonymous SNP can drastically increase the likelihood of an individual developing a disease or condition. Recent research has shown that these silent variations may affect the affinity of RNA binding proteins for mRNA, the splicing of pre-mRNA, and the stability of pre-mRNA [39,40,41]. This, in turn, may compromise the structure of the mRNA, and consequently influence protein formation, efficacy or concentration [42,43]. Once protein function or concentration has been altered, action potential firing in the heart can be disrupted and lead to the development of atrial fibrillation.

### 3.3. Analysis of Exonic SNVs

The exonic SNVs shown in Table 1 were explored further using the NHLBI Exome Sequencing Project (ESP). This project is a collaborative effort between various institutions and research hospitals to use NGS of the human exome to discover new genes and the mechanisms through which they influence the development of heart, blood, and lung disorders. The project expands across diverse populations and facilitates the sharing of conclusions and datasets throughout the scientific community (Exome Variant Server, NHLBI GO Exome Sequencing Project (ESP), Seattle, WA, USA [44]. 11 exonic SNVs in the ESP database were searched. The new noteworthy information that the ESP database yielded for each SNV included variation impact analysis values. This value helps predict the likely impact of an amino acid substitution on protein structure and function [45]. The PolyPhen-2 scores for the variants ranged from benign 0.0 for the SNVs rs6795970, rs180514, rs1805127, rs12621643, and rs1800172—to possibly damaging 0.952 for SNV rs1805123. A score of benign suggests that the amino acid substitutions in each of these SNPs have no serious consequences on protein function. However, rs679570 was associated with time changes in the electrical cycles of the heart and an increased risk of heart block [46]. Conversely, rs846111 was associated with changes in the Q-T interval of the heart [47]. Finally, although rs113180 was not assigned a PolyPhen-2 score, this synonymous SNP is associated with a nearly three times higher likelihood of an individual developing lone atrial fibrillation [38].

### 3.4. Non-Synonymous Variation

Table 1 shows ten nsSNVs, one is a variant in a gene that encodes a ring finger protein, the other nine SNVs are found in genes that encode sodium ion channels or potassium voltage-gated channels. The two sodium ion channel genes are SCN10A and SCN5A. They are the type X and type V voltage-gated sodium channels, respectively. Table 1 shows that the SCN5A gene hosts two nsSNVs. The first is an amino acid substitution from serine to tyrosine at position 1103. The second is an amino acid substitution from histidine to arginine at position 558. The gene SCN10A contains the SNV rs6795970, an A/G base pair polymorphism at chromosome 3 position 38766675. This mutation in the exonic region results in an amino acid change from valine to alanine at position 1073. The specific voltage-gated sodium channel is labeled Nav1.8 and the protein is more commonly known for its role in facilitating cold perception in afferent nociceptive fibers [1]. Despite the association of this SNV with cardiac arrhythmias, the role of the Nav1.8 sodium channel in the electrophysiology of the heart is still uncharacterized [2]. The influence of the amino acid substitution on the function of the voltage-gated sodium channel is still unknown.

The genes found with nsSNVs that code for potassium voltage-gated channels are KCNE1, KCNH2, KCNQ1, and KCNE4. KCNH2 and KCNQ1, found in chromosome 7 and 11 respectively, are genes whose variants have been associated with long QT syndrome [48,49]. In KCNH2, the two nsSNVs are substitutions from lysine to threonine at amino acid positions 897 and 557. In KCNQ1, the variant results in an amino acid substitution from glycine to serine at position 643. The final two genes, KCNE1 and KCNE4 are found on chromosome 21 and 2, respectively. They belong to the KCNE family of genes, which encode trans-membrane proteins with a wide array of functions. Each of these genes contains non-synonymous SNPs that have associations with cardiac arrhythmias [50,51].

The RNF207 gene codes for a ring finger protein and contains the variant rs846111. This variant is found on chromosome 1 position 6279370. RNF207 codes for ring finger protein 207. The protein plays a role in intracellular zinc ion binding [3]. The specific *in vivo* function of the protein is still unknown. Although it is known that the variation translates to a shift of glycine to alanine at position 603, the specific influences of this amino acid substitution on the protein’s function are not yet understood.

### 3.5. Nitrosylation

PTMs such as glycosylation, phosphorylation, and nitrosylation help maintain cardiac rhythm, and modulate gating, localization, and cardiac channel expression levels [12]. To identify additional variations potentially connected to cardiac arrhythmias, we investigated the PTM Nitrosylation. Nitric oxide (NO) is synthesized by a majority of cardiac cell types and plays a vital role in regulating cardiac function [52]. The effects of NO are moderated by S-nitrosylation, which is the covalent modification of a protein cysteine thiol by an NO group to generate an S-nitrosothiol (SNO) [53]. The fundamental roles for S-nitrosylation have been involved in major functions of NO in the cardiovascular system [53].

Gould *et al*. performed a literature search for S-nitrosylated proteins *in vivo* [27]. The 233 S-nitrosylated proteins they compiled display an over-representation of mitochondrial proteins, a significant proportion involved in the generation of precursor metabolites and energy, and pathological conditions associated with an overproduction of NO, which results in inappropriate S-nitroyslation and dysfunction of proteins [27]. To further understand which nitrosylated proteins were affected by variation, we mapped the nitrosylation sites of 233 S-nitrosylated proteins to non-synonymous variations. We found seven cysteines that have loss of S-nitrosylation sites due to nsSNVs (Table 2).

Myosin, a motor protein listed in Table 2, plays a role in regulation of the heartbeat and cardiac function. Studies have shown that mutations of the cardiac myosin gene are the most common cause of inherited hypertrophic cardiomyopathy [54]. Nearly 200 disease-associated myosin mutations have been identified since the discovery of the β cardiac myosin heavy-chain (β-MHC) gene missense mutation’s role in hypertrophic cardiomyopathy [54,55]. β-MHC is a sarcomeric protein expressed in the right and left ventricles of the heart, as well as many skeletal muscles [56]. The precise location of the mutation or alteration of the myosin gene appears to influence the survival of patients; further investigation of this variation may lead to better prognostics for people affected by the mutation [55].

**Figure 1:**
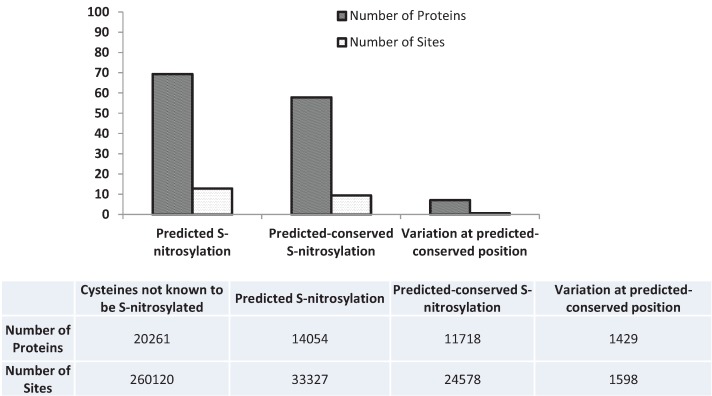
Percentage of predicted S-nitrosylated proteins and sites and the ones which are conserved across mouse, fly, plant or yeast and the nsSNVs mapped to these proteins and sites. Details are available in Table S2.

The current list of experimentally verified nitrosylation sites is not comprehensive. Table S2 contains the list of 1429 proteins which have conserved cysteines across eukaryotic species predicted to be S-nitrosylated and affected by nsSNPs and Figure 1 shows that several of these sites have nsSNVs. To better understand the distribution of predicted S-nitroyslation sites with conserved motifs, we analyzed the dataset to observe if there was over- or under-representation (compared to the complete human proteome) of pathways and functional elements/biological processes. An initial analysis using the UniProtKB/Swiss-Prot keyword ‘Disease’ showed that the keyword was over-represented in the predicted list of S-nitrosylated proteins (observed: 356; expected: 196.96; *p*-value: 3.64E-29). The highly significant p value proved that there are a higher number of disease proteins that are present in Table S2. For several of the known or predicted S-nitrosylation sites that are lost due to nsSNVs we were able to identify diseases associated with these variations (examples—dbSNP id: rs104894204, gene: CSRP3, UniProt AC/position/variation: P50461/58/c->g, disease: familial hypertrophic cardiomyopathy type 12; dbSNP id: rs121909300, gene: OXCT1, UniProt AC/position/variation: P55809/456/c->f, disease: succinyl-coa-3-ketoacid-coa transferase deficiency; dbSNP id: rs121909267, gene: CASR, UniProt AC/position/variation: P41180/131/c->w, disease: familial isolated hypoparathyroidism). S-nitrosylation has not yet been directly connected to these diseases and hence sites such as these (additional ones available in Table S2) are ideal for further biochemical analysis to elucidate the role of S-nitrosylation in cardiac and other diseases.

The PANTHER gene ontology database [57] determined if the predicted loss of S-nitrosylation sites affected by nsSNVs were statistically enriched with a particular molecular function or biological process. Over- and under-representation of PANTHER Pathways, Protein Classification, and Gene Ontology (GO) terms in the predicted list of mutated cysteines, provided an overview of possible effects these non-synonymous variations might have. Table 3 contains the results of the PANTHER statistical overrepresentation test. The major pathway identified as over-represented is blood coagulation (observed: 13; expected: 3.82; *p*-value: 3.64E-29). Atrial Fibrillation, a common type of arrhythmia, can lead to blood clots and other complications. Antithrombotic prophylaxis for stroke, a major risk of atrial fibrillation, is associated with an increased risk of bleeding [58].

The major broad terms for Gene Ontology (GO) Biological Processes that were statistically over-represented were Muscle Contraction, Neurological System Process, and Cellular Organization. For GO Molecular Function, the major terms included receptor activity (includes G-protein coupled receptor activity), motor activity, and structural constituent of cytoskeleton. The cytoskeleton was the top over-represented GO cellular component (*p*-value: 1.09E-03). The cytoskeleton of cardiac myocytes consists of actin filaments, intermediate filaments, and alpha- and beta-tubulin that form the microtubules by polymerization [59]. Studies have shown that the extra-sarcomeric cytoskeleton plays a part in the growth response of the heart and in the pathogenesis of cardiomyopathies [60]. Acquired forms of heart failure have displayed an altered expression of cytoskeletal proteins [61].

Microtubules were also statistically over-represented in GO cellular components (*p*-value: 5.89E-03). Recent studies have investigated the role of microtubules in cardiac arrhythmias. In hypertrophy and heart failure, the accumulation of microtubules disrupts sarcomere motion, contributing to declining ventricular compliance [61]. Microtubule integrity has been linked to cardio-protection, while microtubule disruption has been associated in the response to ischemia in cardiac myocytes [62].

### 3.6. Next-Generation Sequencing and Variation

As the cost of exome sequencing decreases, the use of patient exome sequencing will increase as a diagnostic tool. Here we describe a possible workflow for identifying variation from NGS data using a High-performance Integrated Virtual Environment (HIVE), a cloud-based environment [63].

**Figure 2:**
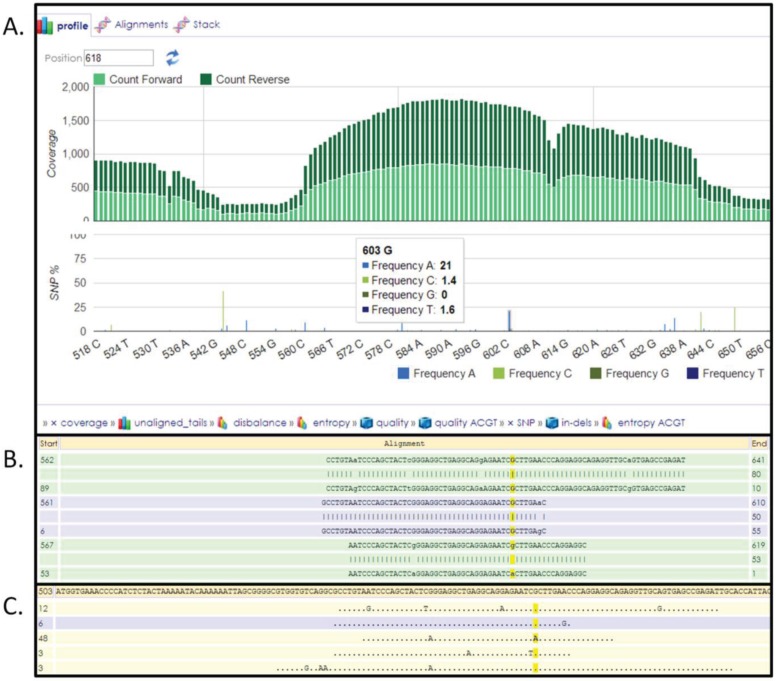
HIVE interface showing results obtained from SNV profiling of human exome reads mapped to FASTA sequence surrounding a SNV. (**A**) Overall coverage result with the 603 position showing variation. (**B**) Reads mapped to the reference with the yellow highlighting the column selected. (**C**) Only variations are shown in this panel.

The FASTA sequences surrounding a SNV are obtained from dbSNP by querying the database using the dnSNP ID from Table S1 followed by “Send to” option and selecting “File” at “Choose Destination” ≥ “FASTA”. This file is then uploaded to HIVE and used as a reference to which NGS data from an individual can be mapped (for additional details see help section on HIVE). Once the input files (FASTA file downloaded from dbSNP and NGS data obtained from an individual) are selected then HIVE-hexagon, a sequence mapping algorithm, is used with default parameters to map the NGS reads to the FASTA record which has the SNV in it. Once the mapping is complete then HIVE-pentagon, a SNV profiling tool, is used to identify all the variations in the individual. Visualization of the mapping results provides an easy overview of the variations present in the individual an example of which is shown in Figure 2.

## 4. Conclusions

We expect that as whole genome sequencing becomes more affordable, genetic testing will play a larger role in the diagnosis of cardiac disorders, and that resources such as Table S1 will become more valuable and common place. In addition to their capacity to sequence whole genomes, the NGS platforms are capable of targeted sequencing. Table S1 also contains nucleotides that are upstream and downstream of each variant. This information allows researchers or physicians to quickly generate libraries of nucleic acids as the primary step in creation of a template. There is currently no quick and easy way to compile a list of variations that have associations with a condition or disease. This table provides a solution for this gap through manual curation. Rather than having physicians and researchers sift through papers to isolate SNPs that are associated with diseases, they can simply create a library of nucleic acids using the information contained in the table. These libraries can then be clonally amplified and their fragments can be prepared specifically per the requirements of the various NGS platforms. The list of these variations is also available from the BioMuta database [64]. It is important to note that DNA variants are rarely the sole determinant of whether an individual will acquire a disease or not. Consequently, it is important that healthcare workers as well as patients remember that these SNVs are a single parameter in a complex biological system. An additional danger that arises from sequencing patients as a diagnostic tool is the incidental discovery of presumed deleterious mutations [65]. 

In an attempt to identify more variants potentially connected to the disease, we investigated the post-translational modification nitrosylation in depth. Many studies have examined potential associations between acquired arrhythmias and post-translational modifications, such as glycosylation, phosphorylation, and nitrosylation. Table 2 and Table 3 provide both experimental and predicted approaches for discovering loss of S-nitrosylation sites that are affected by nsSNVs and details of the amino acid changes are present in Table S2. This information can be used to develop proteomic strategies for variation detection.

## Supplementary Files

Supplementary File 1 Supplementary Materials (ZIP, 312 KB)

## Supplementary Materials

Table S1. Single-nucleotide polymorphisms associated with cardiac arrhythmias extracted from publications.

Table S2. Predicted S-nitrosylation sites, which are conserved in other eukaryotes and have non-synonymous variations.
