# Supplementary material for: Single-Nucleotide Variations in Cardiac Arrhythmias: Prospects for Genomics and Proteomics Based Biomarker Discovery and Diagnostics
Source: Genes (Basel). 2014 Mar 27;5(2):254–69. doi: 10.3390/genes5020254 (PMC4094932; doi:10.3390/genes5020254)
Supplement: Supplementary File 1 — Supplementary Materials (ZIP, 312 KB) [file genes-05-00254-s001.zip › Table S1.pdf]

Supplementary Information

Table S1. Single-nucleotide polymorphisms associated with cardiac arrhythmias extracted from publications.

Gene	Polymorphism	Accession No.	5'-Near sequence	Variant	Wild type	3'-Near sequence	Chr	Position	Amino aicd change	PMID	Year
SCN10A	rs10428132	NG 031891.1	aaggaggtgacaagtgcattgaacacagag	G	T	ttgagaataagtgagctaagtgaagactaa	3	38777554	Intron	23872634	2013
HEY2	rs9388451		cttaatgttcttagtgtgaagacaaaatcc	C	T	tcacctcactacgaggccatgcaagatcca	6	126090377	Intron	23872634	2013
SCN5A	rs11708996	NG 008934.1	ccacactcttacaagctgtgagttcccact	C	G	ttccacaaccctgtcaacacacagaagtct	3	38633923	Intron	23872634	2013
TBX5	rs3825214	NG 007373.1	ctcttgttgcagggctaacaccaccagtca	A	G	gctcatggcagacataattcacaataatac	12	114795443	Intron	23717681	2013
NOS1AP	rs12143842		tgaattagcacccagggtcacatcccagtt	C	T	aaaaatatcccatggagtgcagtcatctca	1	162033890		23166209	2012
ATP1B1	rs1320976	NG 023230.1	ggtctttaccagcttgttctgtgtgatgaa	A	G	tggttagatgagtggtacaattccagatgg	1	169073346		23166209	2012
ZEB1	rs220073	NG 017048.1	tagaaaattttgaaagcaccgagctaaatg	A	C	aatattttcctacaaacctaatgtttcctt	10	31808203	Intron	23130627	2012
	rs13017846		ggcttctcctgcctctgttgtctacagtga	A	G	tatttatggagataaacttccaaacttttc	2	40757791		PMC3397256	2012
	rs6817105		tatttacattaattgatcagttttttatta	C	T	cttttttctggtccttttcttactaatttg	4	111705768		PMC3366038	2012
ZFHX3	rs2106261	NG 013211.1	gggccacttggatattttaatggatggttg	A	G	acaattctctggacgagctctatctgtggg	16	73051620	Intron	PMC3366038	2012
KCNN3	rs6666258	NG 016807.2	gagtaaggatgagatgtgcgagccgaaggg	C	G	ctgtgcagggctggggagagcgggctgagt	1	154814268	Intron	PMC3366038	2012
KCNJ2	rs7219669		aaaggagagaaaaggaagatattgccaagt	G	T	aatgactccatgataaaatcttaaggccaa	17	68521861		22342860	2012
	rs10033464		ttcttttttacattgttagagtcaagaaa	G	T	aagtgctttcatcaagctctgagttacaga	4	111720761		pmc3132749	2011
	rs3853445		ccagttcaagcattttcttagccaagatac	C	T	tcataaaccttgccaagagaaaagggcatg	4	111761487		pmc3132749	2011
	rs6838973		tgagcctccactggaggaatctgtggagta	C	T	ttgctcctcacatttctgtttccaatcgga	4	111765495		pmc3132749	2011
ZFHX3	rs7193343	NG 013211.1	ctaatggcatgtcaattaaaggggtcacca	C	T	aaacaagctgttcaaactttcccctccact	16	73029160	Intron	pmc3132749	2011
KCNN3	rs13376333	NG 016807.2	tcatgggatcggcaggttctgctccttcct	C	T	atctgacaatgccatttaagttctctccag	1	154814353	Intron	pmc3132749	2011
KCNN3	rs1131820	NG 016807.2	acgtgaagtccagctcttcgtgatcgacaa	C	T	ggcgcggatgactggcggatagccatgacc	1	154744852	N44N	21398315	2011
SCN5A	rs3922844	NG 008934.1	ctacctcttcaccttctgtacatattccac	A	G	tgaatatgtattacttttaagccaaaggca	3	38624253	Intron	21347284	2011
ZFHX3	rs2106261	NG 013211.1	gggccacttggatattttaatggatggttg	A	G	acaattctctggacgagctctatctgtggg	16	73051620	Intron	21107608	2010
	rs2824292		caggccatctagaagtccttacaggctctt	A	G	tagcaggcaaggaacaggtgcacagaccaa	21	18787176		20622880	2010
SCN10A	rs6795970	NG 031891.1	caggaagctgacatacctacctcagcaggg	A	G	cctgaggaacagactcatctttccacgtct	3	38766675	V1073A	20062061	2010
	rs7638908		gaaagctaaacttttggagtcgctgtgtag	C	T	cgggtagaggacacggcggcgcgagccgga	3	27754711		19389651	2009
XYLB	rs2070488		gcccgggagcttctcaggtgagagaccatc	A	G	gaatttgtttgtagcatttgcattatgaaa	3	38442490	Intron	19389651	2009
EXOG	rs2284820		caaggaagaaacggtgtgtttttctgaata	C	T	ccaactaggacctgtccctatattagacag	3	38562579	Intron	19389651	2009
EXOG	rs1065800		ggtcttggaggcagcttttagaaatcctct	A	G	tgtagttcctgtccacatgcactatatatt	3	38566480		19389651	2009
ACVR2B	rs2268757	NG 011791.1	gtgcaggttcccttgcctttgggcatgtta	C	T	gatcctgaaaatatgtctaaaagctgggtg	3	38505853	Intron	19389651	2009
ACVR2B	rs1058945	NG 011791.1	agataagctctttgtaacacatttccagtc	A	G	ctaatgctcaaatgtagaacattcctttaa	3	38532511		19389651	2009

Table S1. *Cont.*

Gene	Polymorphism	Accession No.	5'-Near sequence	Variant	Wild type	3'-Near sequence	Chr	Position	Amino aicd change	PMID	Year
XYLB	rs1002676		gtcaccatgtggagcacatccagagctgca	A	C	tatccaatacagtagctgcaagccatgtat	3	38444932	Intron	19389651	2009
EXOG	rs2051215		atagacaagaaaatcaaggctttaaggatc	A	G	agtctcaggcatatagcttataattgtcaa	3	38560345	Intron	19389651	2009
ACVR2B	rs7373828	NG 011791.1	cttccaataagattttgtttgaatatcagc	A	G	cttcaaggcaagtagtggtgtctatcctag	3	38524311	Intron	19389651	2009
XYLB	rs2067082		gtgaacctaaatgggaaacttcaccattat	C	G	cccatgaaacatgctcttgttagcttgggt	3	38438182	Intron	19389651	2009
	rs2461751		aaatgaacaaaatgcccattcatcccagcg	C	T	tcaagagtagtgggggagatagacaattct	2	176289319		19389651	2009
RNF207	rs846111		atatttttcagtagaggctcttctgagagg	C	G	cgttcggagcccagctgtttcctttaggct	1	6279370	G603A	19305409	2009
ATP1B1	rs10919071	NG 023230.1	gtataagtctcagtgagatgaattgtagct	A	G	tactaggtaaaactttttaaaagaacagaa	1	169099483	Intron	19305409	2009
SCN5A	rs11129795	NG 008934.1	tcaaatattatatgattccacttatataag	A	G	catttagggtagttgaatttgtcaaatatt	3	38589163		19305409	2009
PLN	rs11970286		ccagtctgaggccaaaggcatgagaactag	C	T	gaatgggaggaggagtgaaggtaggtgagg	6	118680374		19305409	2009
KCNH2	rs2968863		actgaacttacagccagtccttgtatgaac	A	G	gctttccagtcatttgtgttgtttgaagct	7	150623137		19305409	2009
KCNQ1	rs12296050	NG 008935.1	ccggaaaatgcactgtctctcctgggctcc	C	T	aacctttcacaggccacaggcccattccca	11	2489342	Intron	19305409	2009
LITAF	rs8049607		gccaggctggaaatgggacaaaacccagca	C	T	gcttttttgctttctttttctttgtagag	16	11681753		19305409	2009
NDRG4	rs7188697		agccaccacactccagcctgggtgacgaga	A	G	agagactctgtctcaaaaacacccaaggta	16	58622178	Intron	19305409	2009
KCNJ2	rs17779747		tttccaaaaatatcttgtgaggtccaaatg	G	T	tagccagaattctgtctttgtttgatgagc	17	68494992		19305409	2009
NOS1AP	rs4657178	NG 015979.1	gggtgatatactaagtattggggatttcac	C	T	tggagcaattatggccctgcccttgcagag	1	162210610	Intron	19305409	2009
PLN	rs12210810		cccacagacactgtcctgtgttcacacaga	C	G	gcctggctctggcagaacaaatggtgctat	6	118653204		19305409	2009
	rs10033464		ttcttttttacattgttagagtcaagaaa	G	T	aagtgctttcatcaagctctgagttacaga	4	111720761		19141561	2009
NOS1AP	rs6670339	NG 015979.1	atccaattctggacaaaaatgagctcaagg	C	T	tttggtgctgaaatgaggtcttgcttttca	1	162055806		18927126	2009
NOS1AP	rs1415257	NG 015979.1	atcttaagaaaatccactagctgaaacact	A	G	aacttcctaaaacaaaagaatgaatgaata	1	162062044	Intron	18927126	2009
NOS1AP	rs1415259	NG 015979.1	catttgagaggcaaagctctcaccatccca	A	G	attacatttctagacaattcctcttccaag	1	162085309	Intron	18927126	2009
NOS1AP	rs4657140	NG 01 5979.1	agtgtcatccatgagtttttaaatttgcta	A	T	ttagttgctctgtttattttttgtagagac	1	162061265	Intron	18927126	2009
NOS1AP	rs10494366	NG 015979.1	gttgatcagatatttatgggaggtatgcag	G	T	ttttaaattctgagaatttgtactggtctc	1	162085685	Intron	18927126	2009
	rs2880058		agacattggatcagcatccatctgaattca	A	G	tatgactgcatcttaaaccaattactaatg	1	162014632		18927126	2009
	rs7550692		catacatatacacacatgcatggggtcaac	C	T	ttgggcaaaattgcatatttggaaaagttt	1	162029291		18927126	2009
NOS1AP	rs1932933	NG 015979.1	aaaatacatttgactgagtggaatcctcca	A	G	aaatgaacacagattcaccccaagtgccca	1	162118046	Intron	18927126	2009
	rs4656349	NG 015979.1	ttgtcaggattaaaaatcacagaatcttaa	A	G	tacggtattgtctgtcttgtctttctctga	1	162049824	Intron	18927126	2009
	rs17042171		acagggtgcctgaggacagtggcatagcaa	A	C	tttccagtgaggtaatttaatttgttaaat	4	111708287		PMC2761746	2009

© 2014 by the authors; licensee MDPI, Basel, Switzerland. This article is an open access article distributed under the terms and conditions of the Creative Commons Attribution license (http://creativecommons.org/licenses/by/3.0/).
